# Frequency of dawn phenomenon and its associations with age, HbA1c and diabetes duration in Japanese type 1 diabetes mellitus (T1DM) using the continuous glucose monitoring system (CGMS)

**DOI:** 10.1186/1687-9856-2013-S1-P35

**Published:** 2013-10-03

**Authors:** Ayako Yoshida, Tatsuhiko Urakami, Junichi Suzuki, Misa Okuno, Masako Habu, Remi Kuwabara, Shouri Takahashi, Hideo Mugishima

**Affiliations:** 1 Dept. of Pediatrics, Nihon University School of Medicine, Tokyo, Japan

## Aims

We defined the dawn phenomenon is an increase in either insulin requirements or the plasma glucose concentration, in the absence of preceding hypoglycemia or waning insulin levels, occurring between the hours of 0400 and 0800. According to previous studies, the frequency of dawn phenomenon is reported to be approximately 54.0% inpatients with type 1 diabetes mellitus (T1DM),and the poor glycemic control is associated with an exaggerated dawn phenomenon.The present studies were undertaken to assess the associations between dawn phenomenon and age at the time of the study, HbA1c and diabetes duration in Japanese T1DM showing the dawn phenomenon.

## Methods

Study subjects consisted of 21 patients (3 males and 18 females)with T1DM who had a duration of more than half a year based on CGM. The mean age was 22.1±15.9yr; diabetes duration 11.1± 9.7 yr; HbA1c 8.3±2.2 % ( JDS ). All subjects were receiving therapeutic injections of insulin, 4 were managed by multiple daily injections (MDI) and 17 with continuous subcutaneous insulin infusion (CSII).

## Results

The dawn phenomenon was present in 8 of the 21 patients (38.1 %). The plasma glucose concentrations increased a mean of 69.5% from the overnight nadir to the pre-breakfast time point. The patients with dawn phenomenon were compared in terms of diabetes duration (13.0 ± 9.9 vs 10.0 ± 9.7: p<0.01)yr , HbA1c(8.3 ± 1.6 vs 8.5 ± 2.4: p<0.01)%, age at the time of the study (24.6 ± 18.0 vs 20.5 ± 14.9: p<0.01)yr. The subjects with dawn phenomenon had longer diabetes duration, lower HbA1c and were older. Furthermore, these subjects experienced hypoglycemia (< 70mg/dl) during the daytime.

## Conclusion

The frequency of dawn phenomenon in the present study was lower than that in the previous studies .This might be attributable to there being many users of CSII amongour subjects and to Japanese foodscontaining a large amount of the carbohydrate as compared with protein. The associations of dawn phenomenon with longer diabetes duration and advanced age may be based on poor glycemic control. Furthermore, excess bolusescause hypoglycemia and low HbA1c.These results suggest that change in the basal insulin rate should be considered instead of an increase in the pre-meal bolus. We conclude that CGM should be used to adjust CSII.

